# To: Delirium and sleep quality in the intensive care unit: the role of melatonin

**DOI:** 10.62675/2965-2774.20250280

**Published:** 2025-05-28

**Authors:** Josef Finsterer, Sounira Mehri

**Affiliations:** 1 Neurology & Neurophysiology Center Vienna Austria Neurology & Neurophysiology Center - Vienna, Austria.; 2 "Nutrition-Functional Foods and Vascular Health" Biochemistry Laboratory Faculty of Medicine Monastir Tunisia Biochemistry Laboratory, "Nutrition-Functional Foods and Vascular Health", Faculty of Medicine - Monastir, Tunisia.

To the Editor

The interesting article by Soares et al. on *delirium* and sleep hygiene in the intensive care unit (ICU) and the role of melatonin^(1)^ raises concerns that should be discussed.

The first point is that sleep hygiene and the risk of *delirium* in the ICU depend not only on age, comorbidities, disease severity, environment, and therapeutic interventions,^(1)^ but also on the type of ICU, reason for admission, history, and genetic factors. Regarding the ICU type, the risk of sleep deprivation or *delirium* may depend primarily on whether the patient is, for example, in a neurological ICU or a cardiac ICU. Patients with cerebral disease are more likely to experience sleep disturbances and develop *delirium* compared to patients in a cardiac ICU. Patients with a history of psychiatric disease (e.g., depressive or psychotic episodes, social withdrawal, Hikikamori) may be at higher risk of poor sleep quality or *delirium* in the ICU than patients without. Patients with hereditary hyperammonemia, trisomy 21, spinocerebellar ataxia, Niemann-Pick disease, and several other genetic disorders may be at higher risk of developing *delirium* or sleep deprivation than patients without a genetic disease.^(2)^

The second point is that sleep can be disturbed not only by the external influences of light and noise but also by temperature, humidity, diet, fluid intake, number of probes attached to the patient, and the amount of electrosmog.^(3)^ Since ICUs are equipped with numerous electronic devices and nurses, doctors, and patients carry their mobile phones, laptops, and tablets with them 24 hours a day, everyone in the ICU, including patients, is exposed to these external stressors, which can inevitably and severely affect biorhythms. Catheters, probes, and sensors attached to the patient can prevent relaxation due to permanent haptic disturbances.

The third point is that internal causes of sleep deprivation, such as overstimulation due to increased secretion of cortisol or adrenaline, can lead to patients being unable to fall asleep and stay asleep. Stress with the consecutive secretion of stress hormones can arise not only from external stressors but also from internal stress factors, such as pain, fear, insecurity, depression, hopelessness, unpleasant physical sensations, discomfort with the situation, delusions, or lack of information about the current situation, and being torn away from familiar surroundings.

A fourth point that has not been considered as affecting sleep quality and the likelihood of developing *delirium* is medication withdrawal. There is a high risk of sleep deprivation and *delirium*, particularly in patients weaned from mechanical ventilation. Withdrawal of analgesics, sedatives (especially benzodiazepines), relaxants, antiepileptics, hypnotics, or adrenergics can result in withdrawal syndromes, which in turn lead to sleep disorders and *delirium*.

The fifth point is that *delirium* can be easily missed in the ICU.^(4)^ Particularly in sedated and relaxed patients, the typical manifestations of *delirium* may not occur unless mechanical ventilation, sedation, and muscle relaxation are stopped. Even more challenging to diagnose is hypoactive *delirium*, in which there are no motor symptoms or hyperactivity but vegetative overstimulation, which nevertheless leads to disorientation and confusion.

The sixth point is that hyperactive or hypoactive *delirium* is a severe psychiatric disease that should not be treated with melatonin but with neuroleptics (e.g., risperidone, haloperidol) and, if necessary, benzodiazepines or propofol.^(5,6)^

It is highly questionable whether improving sleep hygiene with melatonin can prevent the development of *delirium*. All external and internal risk factors must be eliminated to prevent delirium, and if *delirium* becomes manifest, it must be treated acutely by a psychiatrist. *Delirium* is an emergency.
